# Benign Tumors of the Esophagus: A Histopathologic Study of 49 Cases among 931 Consecutive Esophageal Biopsies

**DOI:** 10.4021/gr2009.02.1277

**Published:** 2009-03-20

**Authors:** Tadashi Terada

**Affiliations:** Department of Pathology, Shizuoka City Shimizu Hospital, Miyakami 1231 Shimizu-Ku, Shizuoka 424-8636, Japan. Email: piyo0111jp@yahoo.co.jp

**Keywords:** Esophagus, Benign tumor, Clinicopathology, Immunohistochemistry

## Abstract

The author reviewed 931 consecutive esophageal biopsies in the last 15 year in out pathology laboratory of our hospital in search for benign esophageal tumors. As the results, 41 cases (4.4%) of squamous papilloma, 4 cases (0.4%) of granular cell tumor, 3 cases (0.3%) of leiomyoima, and 1 case (0.1%) tubular adenoma were identified. The 41 cases of squamous papillma were located in the cervical esophagus in 6 cases, in the proximal esophagus in 12 cases, in the middle esophagus in 11 cases, and in the distal esophagus in 12 cases. The squamous papilloma was immunohistochemically positive for various cytokeratins. It was endoscopically recognized as small polypoid tumor. The age ranged from 35 years to 81 years with a mean of 51 years. Male to female ratio was 25:16. The 4 cases of granular cell tumor were located in the proximal esophagus in 3 cases, and in the middle esophagus in 1 case. The granular cell tumor was immunohistochemically positive for vimentin, S100 protein, and neuron-specific enolase. It was endoscopically recognized by elevated small lesions. The ages of were 36, 45, 67 and 78 years, and male to female ratio was 1:3. The 1 case of tubular adenoma was located in the distal esophagus. Histologically, it was associated with heterotopic gastric mcusa, and immunohistochemically faintly positive for p53 protein and Ki-67 antigen. It was endoscopically a slightly elevated lesion. The patient was 46 year-old man. The 3 cases of leiomyoma were located in the cervical esophagus in 1 case and in the proximal esophagus in 2 cases. It was immunohistochemically positive for vimentin, α-smooth muscle actin, and desmin. It was endoscopically recognized as a submucosal tumor. The ages were 34, 45, and 85 years. Male to female ratio was 1:2.

## Introduction

Many kinds of benign pathologic lesions occur in the esophagus. They include esophageal atresia, heterotopic gastric mucosa, heterotopic pancreatic tissue, diverticula, esophageal cyst, achalasia, Lye stricture, reflex esophagitis, Barrett’s esophagus, herpes simplex esophagistis, cytomegalovirus esophagitis, eosiphophilic esophagitis, Crohn’s disease, candidiasis, leiomyoma, glycogenic acanthosis, amyloidosis, squamous papilloma, hyperplastic polyp, and granular cell tumor [1, 2]. Recent advances in endoscopy have made it possible that these esophageal benign lesions are biopsied and diagnosed correctly. In the present study, the authors reviewed 931 archival cases of the esophageal biopsies in search for benign tumors of the esophagus.

## Materials and Methods

The authors retrospectively reviewed consecutive 931 cases of esophageal biopsy specimens in the last 15 years in the pathology laboratory in our hospital. The ages of the patients ranged from 12 years to 95 years with a mean of 53 years. Male to female ratio was 547:384. Clinical and endoscopic records were also reviewed.

Histochemical stainings including PAS and alcian blue were employed in appropriate cases. In appropriated cases, an immunohistochimical study was performed, using Dako Envision method (Dako Corp., Glostrup, Denmark), as previously described [3, 4]. The antibodies employed were anti-cytokeratin (AE1/3, Dako), anti-cytokeratin (polyclonal wide, Dako), anti-p53 protein (DO-7, Dako), anti-Ki-67 antigen (MIB-1, Dako), neuron-specific enolase (BBS/NC/VI-H14, Dako), CD34 (QBEND10, Dako), vimentin (Vim 3B4, Dako), desmin (D33, Dako), α-smooth muscle actin (1A4, Dako), S100 protein (polyclonal, Dako) KIT (polyclonal, Dako), and PDGFRA (Santa Cruz, CA, USA).

## Results

Forty-one cases (4.4%) of squamous papilloma, 4 cases (0.4%) of granular cell tumor, 3 cases (0.3%) of leiomyoima, and 1 case (0.1%) tubular adenoma were identified.

**Figure 1:**
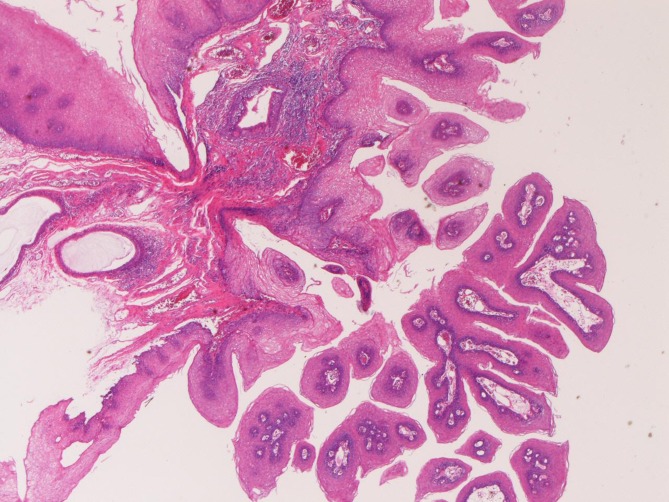
Squamous papilloma of the esophagus. Papillary proliferation of mature squamous epithelium is seen. HE, x20

In the 41 cases of squamous papilloma, the age ranged from 35 years to 81 years with a mean of 51 years. Male to female ratio was 25:16. The presenting symptoms were asymptomatic in 32 cases, nausea in 4 cases, chest burn in 3 cases, and dysphasia in 2 cases. The locations of the 41 cases of squamous papilloma were the cervical esophagus in 6 cases, the proximal esophagus in 12 cases, the middle esophagus in 11 cases, and the distal esophagus in 12 cases. Squamous papilloma was endoscopically recognized as small polypoid tumor. The size of squamous pailloma ranged from 2 mm to 10 mm. Histologically, squamous papilloma was characterized in papillary proliferation of mature squamous epithelium (Fig 1). No koilocytosis was identified in this series. The squamous papilloma was immunohistochemically positive for various cytokeratins and negative for vimentin and other antigens examined.

**Figure 2:**
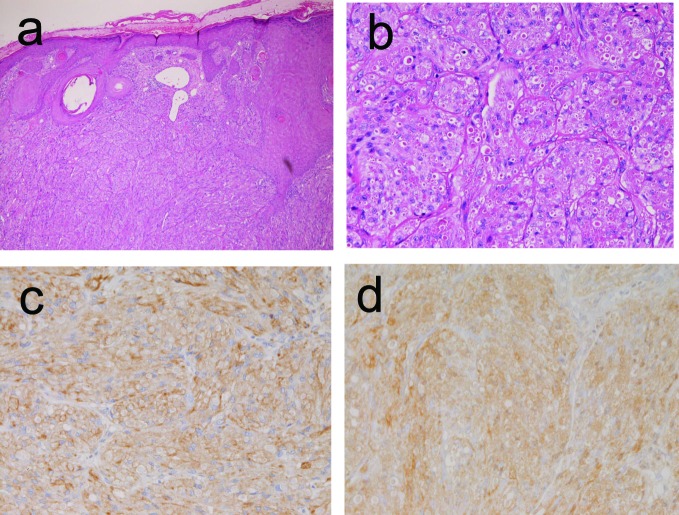
Granular cell tumor of the esophagus. A: Histologic features. Large cells with acidophilic granular cytoplasm are seen. Many hyaline globules are also seen. HE, x200 B: The cytoplasm and hyaline globules are positive with PAS stain. PAS, x200 C: The tumor cells are positive for S100 protein. Immunostaining, x200 D: The tumor cells are positive for neuron-specific enolase. Immunostaining, x200.

In the 4 cases of granular cell tumor, the ages of the patients were 36, 45, 67 and 78 years, and male to female ratio was 1:3. Two cases complained of dysphagia and two cases were asymptomatic. They were located in the proximal esophagus in 3 cases, and in the middle esophagus in 1 case. Granular cell tumor was endoscopically recognized by an elevated small lesion. The size ranged from 4 mm to 12 mm. Histologically, granular cell tumors were characterized by a proliferation of large cells with acidphilic granular cytoplasm (Fig 2a). Hyaline globules positive with PAS stains were recognized in many areas (Fig 2b). The granular cell tumors were immunohistochemically positive for vimentin, S100 protein (Fig 2c), and neuron-specific enolase (Figure 2d), and negative for cytokeratins and other antigens examined.

**Figure 3:**
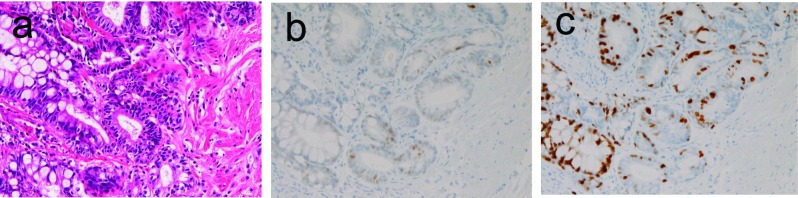
Tubular adenoma of the esophagus. A: adenomatous tubules are seen. HE, x200 B: The tumor cells are very focally positive for p53 protein. Immunostaining, x100 C: The Ki-67 labeling is 26%. Immunostaining (MIB1), x200

In the 1 case of tubular adenoma, the patient was 46 years old man. He was asymptomatic. The tubular adenoma was located in the distal esophagus. It was endoscopically a slightly elevated lesion. Histologically, it was composed of adenomatous proliferation (Fig 3a) reminiscent of gastric or colonic adenoma. Interestingly, it was associated with heterotopic gastric mucosa. Immunohistochemically, it was faintly positive for p53 protein (Fig 3b). The Ki-67 labeling was 26% (Fig 3c).

**Figure 4:**
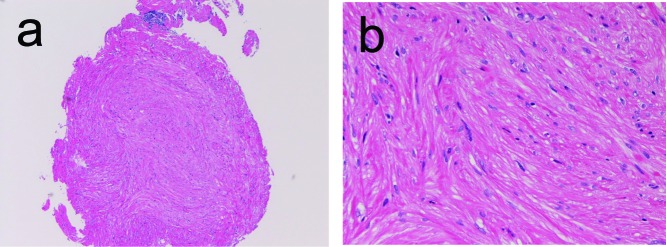
Leiomyoma of the esophagus. A: Low power view shows well-define nodule composed of acidophilic spindle cells. HE, x40 B: The tumor is composed of spindle cells considered as smooth muscle cells. HE, x200

In the 3 cases of leiomyoma, the ages were 34, 45, and 85 years. Male to female ratio was 1:2. The leiomyoma were located in the cervical esophagus in 1 case and in the proximal esophagus in 2 cases. It was endoscopically recognized as a submucosal tumor. The sizes were 4mm, 6mm, and 12mm. Histologically, leiomyoma was characterized by well defined nodule (Fig 4a), composed of spindle smooth muscles (Figure 4b). No atypia or mitotic figures were identified. The leiomyoma was immunohistochemically positive for vimentin, α-smooth muscle actin, and desmin (1 case), but negative for cytokeratins, CD34, KIT, and PDGFRA.

## Discussion

Benign tumors of the esophagus are rare, and most of them are not clinically important lesions. However, differential diagnosis is important. In addition, large benign tumors may obstruct the esophageal lumen, and treatment was difficult in such cases.

Squamous papilloma of the esophagus is a rare lesion. Most of the previously reported study was case reports and studies of small series [5-9]. The squamous papilloma of the esophagus does not transform into squamous cell carcinoma. In the present study, the frequency was 4.4%, and the size was small. The hot debate is whether or not the squamous cell carcinoma is caused by human papilloma virus. Some researchers insisted the association of squamous papilloma and human papilloma virus [5, 7, 8], but others denied the association [6]. In the present study, although no viral examination was performed, koilocytosis as seen in uterine cervix neoplasm and condyloma acuminatum was not recognized.

Granular cell tumor of the esophagus is extremely rare. Only several case reports or studies of small series have been published [10-12]. Its important point is differential diagnosis. In general, acidophilic granular cytoplasm and immunoreactions for vimentin, S100 protein, and neuron-specific enolase are important points in this tumor. The present 4 cases fulfilled the diagnostic criteria of granular cell tumor. The frequency in the present study was 0.4%.

Tubular adenoma of the esophagus is extremely rare. Only a few case reports are present in the literature [13]. In the present study, it is very interesting that the tubular adenoma was associated with heterotopic gastric mcuosa [14, 15].

Adenocarcinoma very infrequently develops in the heterotopic gastric mucosa [16, 17]. However, adenoma developing in heterotopic gastric mucosa has not been reported, to the author’s best knowledge. In the present study, it frequency was 0.1%.

Three leiomyomas were recognized in the present study. Esophageal leiomyoma is rare but the most common mesenchymal tumor. In the literature, several case reports are recorded [18, 19]. Large leiomyoma may obstruct the esophageal lumen, causing serious problems. The most important points of esophageal leiomyoma are differential diagnosis from leiomyosarcoma and gastrointestinal stromal tumor (GIST). In making a diagnosis of leiomyoma, mitotic counts, cellular atypia, and necrosis were required. In the present case, no atypia or mitotic figures were present. Leiomyoma must be distinguished from GIST. In making a diagnosis of GIST, immunohistochemical demonstration of KIT and/or CD 34 is mandatory [20, 21]. In addition, genetic analysis of KIT and PDGFRA genes may be required in KIT-negative GIST cases. The present cases were negative for KIT, CD34, and PDGFRA. Therefore the present cases are true leiomyomas.
